# Porous polylactic acid fibers synthesized by centrifugal spinning with phase separation for oil removal application†

**DOI:** 10.1039/d4ra08727e

**Published:** 2025-04-15

**Authors:** Kenji Kinashi, Masaki Negoro, Hoan Ngoc Doan, Phu Phong Vo, Khanh Van Thi Khuat, Wataru Sakai, Naoto Tsutsumi

**Affiliations:** a Faculty of Materials Science and Engineering, Kyoto Institute of Technology Matsugasaki, Sakyo Kyoto 606-8585 Japan kinashi@kit.ac.jp; b Master's Program of Innovative Materials, Kyoto Institute of Technology Matsugasaki, Sakyo 606-8585 Kyoto Japan; c Tissue Engineering and Regenerative Medicine Laboratory, School of Biomedical Engineering, International University Ho Chi Minh City Vietnam dnhoan@hcmiu.edu.vn; d Vietnam National University Ho Chi Minh City Vietnam; e Faculty of Chemistry, University of Science Ho Chi Minh City Vietnam; f Doctor's Program of Materials Chemistry, Graduate School of Science and Technology, Kyoto Institute of Technology Matsugasaki, Sakyo Kyoto 606-8585 Japan

## Abstract

The development of environmentally friendly oil-absorbing fibrous materials is crucial, as conventional separation materials contribute to secondary pollution due to their nondegradability. In this study, highly hydrophobic and superoleophilic porous polylactic acid (PLA) fibers were fabricated *via* centrifugal spinning combined with nonsolvent-induced phase separation. The fiber porosity was controlled by adjusting the ratio of good solvents to nonsolvent in the spinning solution. The morphology and physical properties of the PLA fibers were systematically analyzed. Among the prepared samples, PLA fibrous membranes spun from a chloroform/dimethylformamide (80/20 w/w) solution exhibited a high water contact angle and superior oil absorption capacity. These results demonstrate the potential of porous PLA fibers as sustainable materials for environmental applications.

## Abstract 2

This study presents porous, hydrophobic, and superoleophilic polylactic acid (PLA) fibers fabricated using centrifugal spinning and nonsolvent-induced phase separation. PLA fibers from a chloroform/dimethylformamide (80/20) solution showed excellent water repellence and oil absorption.

## Graphical abstract

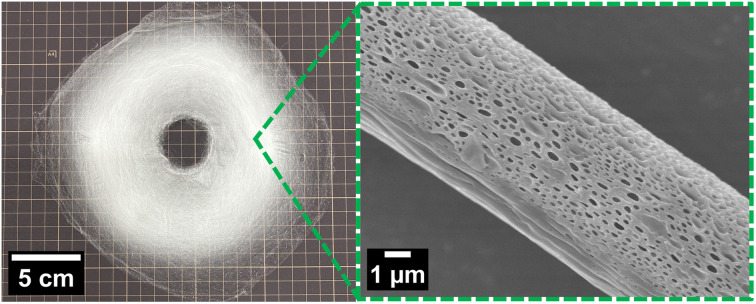

## 1. Introduction

Polymeric fibers, which have fiber diameters ranging from micro to nanoscale, are attractive owing to their high surface area to volume ratio, high porosity, and high flexibility. The excellent properties of fiber materials can offer significant potential in multiple applications such as oil absorbents,^1^ thermal insulators,^2^ and wound dressings.^3^ Phase separation,^4^ self-assembly,^5^ and electrospinning^6^ have so far been commonly used as typical methods for obtaining submicron- and nano-sized fibers. Electrospinning is widely recognized as generating ultrafine fibers due to its facile setup and procedure. In electrospinning, micro/nanofibers are fabricated using high voltage to induce ejection and elongation of a spinning solution by electrostatic repulsions. Although micro/nanofibers can be obtained with simple processing, electrospinning has several drawbacks, such as low productivity and high voltage requirements.

Furthermore, the requirement of spinning solution conductivity limits their development to practical application. As an alternative to electrospinning, centrifugal spinning has attracted attention owing to its high production rate. Centrifugal force is used in centrifugal spinning to draw and stretch polymer jets, allowing a wide range of polymer choices. In addition, the fiber productivity of centrifugal spinning is greater than that of electrospinning.^7^ Therefore, centrifugal spinning has received attention in recent years, and several polymeric fibers have been successfully produced by the centrifugal spinning process.^2,8–11^

Oil pollution has attracted attention because the spilled oil could cause severe impacts on the marine environment. Various techniques for oil spill remediation, including dispersion, burning, and oil absorbents, have been developed.^12,13^ Among them, oil absorbents using synthetic fibers produced from hydrophobic polymers have been widely studied.^14,15^ However, plastic pollution has also recently become alarming worldwide, and microfibers are one of the most prevalent contamination sources of plastic pollution.^16^ Biodegradable materials should be employed to prepare the fibers to reduce the potential pollution from the disposal of the fiber materials after use. As an environmentally friendly polymer, polylactic acid (PLA), synthesized by the ring-opening polymerization of lactide, is widely used in various applications such as wound dressings, scaffold materials, and packaging materials due to its biodegradable and biocompatible properties. Furthermore, with its natural hydrophobicity and biodegradable properties, PLA can be used as a green material for oil sorption applications.

Designing a porous structure on fibers can provide a larger surface area, broadening the applications, especially for oil removal applications. Fabrication methods of porous fibers can be categorized into two techniques: selective dissolution and phase separation. A two-step process achieves porous fiber creation using a selective dissolution technique. Initially, fibers composed of a polymer and sacrificial phase (an immiscible polymer or particles) are prepared. Afterward, sacrificial phases are removed from the fibers selectively to convert the phase to pores. On the other hand, phase separation is an effortless and more straightforward approach for porous fiber fabrication. By controlling the solvent composition of the polymer solution, the nonsolvent-induced phase separation process can be used to obtain porous fibers.^17^

In previous studies, significant efforts have been made to develop PLA fibers using centrifugal spinning. Golecki *et al.* investigated the effect of solvent evaporation on the morphology of PLA fibers and the correlation of Rayleigh instability in the formation of beads.^18^ Patlan *et al.* evaluated the effect of solvent composition (CHCl_3_/DMF) on the morphology and thermal properties of PLA and PLA/carbon nanotube composites.^19^ However, the formation of the porous structure was not investigated in the research conducted. Zhang *et al.* also developed porous PLA fibers using the centrifugal spinning technique and applied them for oil absorption.^20,21^ The researchers reported that porous fibers could be obtained using only CHCl_3_. However, the formation mechanism and the porous structure were not profoundly investigated. Although the effect of solvent and spinning parameters on the morphology of centrifugally spun PLA fibers was investigated in previous studies, the influence of solvent composition on the surface and porous structure has yet to be carefully evaluated. Therefore, this study evaluated the influence of CHCl_3_/DMF ratios in solvent mixtures on the morphology, porous structure, and thermal properties of the PLA fibers fabricated using the centrifugal spinning technique. Furthermore, the oil sorption performance of the prepared porous fibers was investigated to illustrate the potential applications of the centrifugally spun porous PLA fibers.

## 2. Experimental section

### 2.1 Materials

Polylactic acid (PLA) pellets used in this study were Lacty 2012, supplied by Shimadzu Co. (Japan). chloroform (CHCl_3_) and *N*,*N*-dimethylformamide (DMF) were purchased from Waka Co. (Japan) and used as received. All of the materials were used as received without further purification.

### 2.2 Fiber fabrication

PLA/CHCl_3_ solutions with varying PLA concentrations of 6, 8, and 10 wt% were prepared by dissolving PLA pellets in CHCl_3_ using a planetary centrifugal mixer (ARE-310, Thinky Co., Japan) for 15 min at 2000 rpm and degassed for 1.5 min at 2200 rpm. PLA/CHCl_3_/DMF solutions were prepared by dissolving PLA pellets in CHCl_3_ first, then mixed with DMF to give 10 wt% solutions. The CHCl_3_/DMF ratios were fixed at 100/0, 95/5, 90/10, 85/15 and 80/20 w/w. PLA fibers were fabricated from the prepared solutions using a centrifugal spinning apparatus designed in our laboratory. The prepared PLA solution was placed in a syringe and operated using a syringe pump (KDS-100, KD Scientific Inc., Massachusetts, USA) to flow the solution at 70 mL h^−1^. The PLA solution loaded in the syringe was fed to a spinneret *via* a polypropylene tube connected to the syringe. Two needles with an inner diameter of 160 μm and shaft length of 10 mm were equipped with the spinneret, and an AC motor rotated the spinneret at a rotational speed of 15 000 rpm. The distance between the nozzle and the collector was 10 cm. The relative humidity and temperature were kept at 50 ± 5% and 23.5 ± 0.7 °C, respectively. Those parameters were recorded by a hygrothermograph placed in the centrifugal spinning chamber. In our experimental design, the CHCl_3_/DMF solvent ratios were chosen according to literature^22^ to examine how nonsolvent-induced phase separation affects fiber morphology and porosity. Ratios of 100/0, 95/5, 90/10, 85/15, and 80/20 (w/w) were chosen to systematically investigate the influence of increasing DMF content on fiber diameter, pore formation, and surface structure. The samples prepared from the PLA dissolved in a mixture of CHCl_3_ and DMF were denoted as PLA_X, where X denotes the DMF ratio compared to CHCl_3_. For example, PLA_20 means the one prepared from PLA dissolved in CHCl_3_/DMF ratio at 80/20 w/w.

### 2.3 Characterization

The morphology of the fabricated PLA fibers was observed by field emission scanning electron microscopy (FE-SEM) (JEOL-7600, JEOL Ltd., Japan). ImageJ software was used to determine the average diameters of the fabricated PLA fibers, which were determined from 200 fiber diameters. The average fiber diameter and standard deviation were calculated to ensure accuracy and statistical relevance. The average viscosity of liquids used in this study was measured at least three times using a vibro viscometer (SV-1A, A&D Co., Japan) at room temperature. The surface area of the prepared PLA fibrous membranes was measured by a surface area analyzer (Flowsorb II 2300, Micrometrics Instrument Co., USA). The X-ray diffraction (XRD) pattern of the centrifugally spun PLA fibers was evaluated using X-ray diffraction (XRD, SmartLab, Rigaku Co. Ltd., Japan). Cu-Kα (40 kV and 30 mA) was used as the X-ray source of the instrument. The 2-theta scanning range of the XRD measurement was from 5 to 40° at room temperature. The thermal properties of the obtained centrifugally spun PLA fibers were characterized using a differential scanning calorimeter (DSC) (TA Q200, TA Instruments Japan Inc., Japan) and a thermogravimetric analysis (TGA) (Discovery TGA, TA Instruments, USA). The DSC and TGA analyses were performed with a 10 °C min^−1^ heating rate under a nitrogen atmosphere. The wettability of the resulting PLA fibrous membranes was evaluated using a contact angle goniometer (Phoenix 300, Kromtek Co., South Korea). The contact angle of the PLA fibrous membrane was calculated using ImageJ software. The contact angle of each PLA fibrous membrane was determined from five measurements. The tensile test was performed using a (TENSILON RTF-1210, A&D Co., Japan) to evaluate the mechanical stability of the fibrous membranes. The sample specimen, with a gauge length of 10 mm, was subjected to the tensile test with a crosshead speed of 1 mm s^−1^. The oil sorption test determined the maximum oil sorption capacity. The fibrous membrane was cut into a 20 × 20 mm^2^ square specimen, and the specimen was placed in a glass beaker filled with 100 mL of oil. After 60 min, the specimen was picked up from the oil and drained the oil for 3 min. The oil sorption capacity of all sorbents was determined using the following eqn (1):1
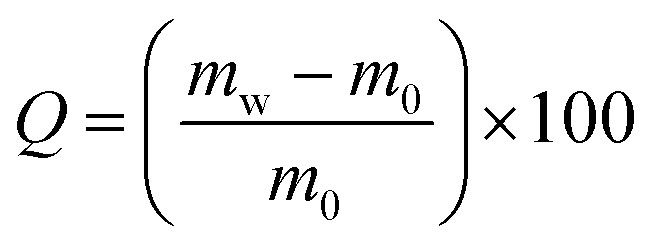
where *Q* is the sorption capacity (g g^−1^), *m*_w_ is the weight of the wet sorbent after 3 min of drainage (g), and *m*_0_ is the initial weight of the dry sorbent. Figure of Merit (FoM) was also evaluated to characterize the comprehensive oil sorption efficiency using the following equation:

2 FoM = *P* × *Q*

where *P* is the production efficiency of fiber (g min^−1^).

## 3. Results and discussion

### 3.1 Morphology and porous structure of PLA fibers

**Fig. 1:**
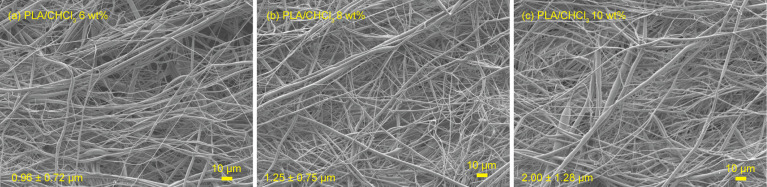
FE-SEM images of the PLA fibers obtained from the PLA/CHCl_3_ solutions with various PLA concentrations of (a) 6 wt%, (b) 8 wt%, and (c) 10 wt%.

In the centrifugal spinning process, various parameters such as solution concentration, rotational speed, evaporation rate, nozzle diameter, nozzle-collector distance, and humidity affect fiber morphology.^9,10,18^ Among these parameters, solution concentration is a key in determining resultant fiber morphology. Thus, the morphology of the centrifugally spun PLA fibers obtained from several solution concentrations was observed before the fiber fabrication from PLA/CHCl_3_/DMF systems. FE-SEM images of the centrifugally spun PLA fibers obtained from 6, 8, and 10 wt% PLA/CHCl_3_ solutions are shown in Fig. 1. PLA fibers were successfully obtained from PLA/CHCl_3_ solutions with PLA concentrations in the 6 to 10 wt% range, as shown in Fig. 1. The FE-SEM observation revealed that higher PLA concentration offers thicker fiber diameters. The measured diameters were 0.96 ± 0.72, 1.25 ± 0.75, and 2.00 ± 1.28 μm when the PLA concentrations were 6, 8, and 10 wt%, respectively. The increase in the PLA fiber diameter with the increase in PLA concentration is attributed to the higher viscosity of the prepared solution. During centrifugal spinning, a polymer jet ejected from a spinneret is elongated by centrifugal force. Higher viscosity resists the centrifugal force, thus preventing the elongation of the polymer jet. The prevented elongation induced by higher PLA concentration offers a thicker fiber diameter. Based on stable spinnability, PLA concentration at 10 wt% was selected as the optimal spinning condition for preparing fibers from PLA/CHCl_3_/DMF systems. Furthermore, the PLA concentration was set at 10 wt% because concentrations lower than 8 wt% result in insufficient fiber formation, while concentrations exceeding 10 wt% lead to excessively high solution viscosity, making fiber spinning challenging. Previous studies have reported that the optimal concentration range for electrospinning PLA fibers is approximately 6–12 wt%,^23^ and a similar trend was observed for centrifugal spinning in this study.

**Fig. 2:**
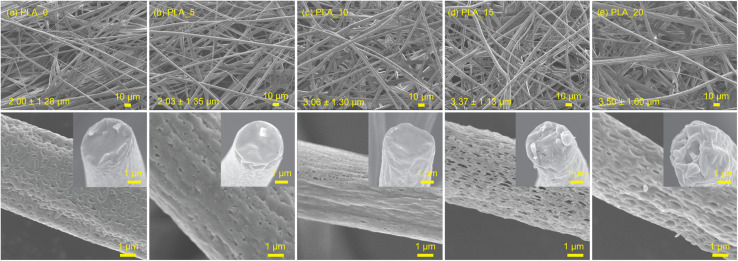
FE-SEM images of surface morphology and cross-section of the PLA fibers obtained from the 10 wt% PLA solutions with various CHCl_3_/DMF ratios of (a) PLA_0, (b) PLA_5, (c) PLA_10, (d) PLA_15, and (e) PLA_20.

The centrifugal spinning of the PLA/CHCl_3_/DMF systems also successfully generated a fibrous membrane. The FE-SEM images of the PLA fibers spun from 10 wt% PLA solution with different CHCl_3_/DMF weight ratios are shown in Fig. 2, and fiber diameter distributions are exhibited in Fig. S1.† Centrifugal spinning could obtain PLA fibers from PLA solution with a CHCl_3_/DMF weight ratio of 95/5, 90/10, 85/15, and 80/20. Although the higher DMF ratio in the spinning solution offered lower viscosity, as shown in Fig. S2,† the fiber diameter of the PLA fibers was increased with increasing DMF ratios. In general, using a solvent mixture with a lower evaporation rate leads to the formation of thinner fibers due to the longer elongation time.^18^ However, the fiber diameter of the PLA fibers increased with an increasing low volatile DMF ratio. The tendency could be assigned to phase separation during the centrifugal spinning process. CHCl_3_ evaporates faster than DMF due to its high vapor pressure compared to DMF, and an outer skin structure is formed. DMF remains due to its lower evaporation rate than CHCl_3_, and the remaining DMF cannot stretch the ejected PLA polymer jet as a nonsolvent for PLA. Therefore, the fiber diameter of the PLA fibers spun from PLA, which is dissolved in a mixture of CHCl_3_ and DMF solvents, was increased with an increase in the DMF weight ratio.

**Fig. 3:**
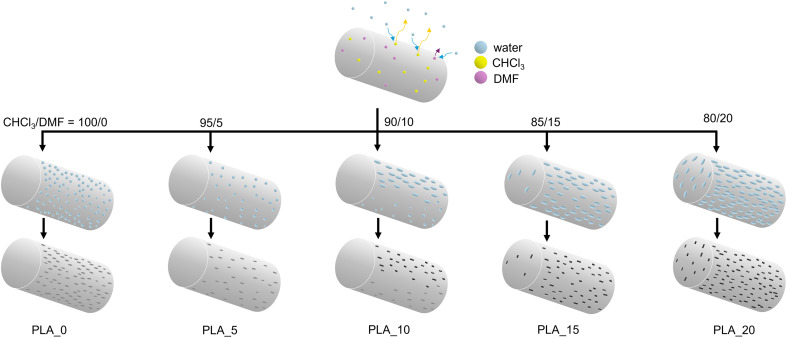
Schematic representation of the solution jet evolution process during centrifugal spinning.

The surface morphology and cross-sectional structures of PLA fibers obtained from different spinning solutions are shown in Fig. 2. In the PLA_0 fibers, pores were observed on the fiber surfaces, while the internal structure remained dense. This phenomenon can be attributed to the breath figure formation caused by the rapid evaporation of CHCl_3_, which possesses a high vapor pressure.^24^ The endothermic evaporation of CHCl_3_ absorbs heat from the atmosphere surrounding the polymer jet, leading to moisture condensation on the jet surface and subsequently initiating the breath figure process. These condensed water droplets eventually evaporate, leaving behind pores on the fiber surface. In contrast, when the DMF ratio in the spinning solution increased, larger surface pores and internal pores were observed within the fibers. This pore formation can be explained by the nonsolvent-induced phase separation (NIPS) mechanism. During centrifugal spinning, the rapid evaporation of CHCl_3_ causes a sudden increase in the polymer concentration and leads to the formation of a dense skin layer on the surface of the jet. Meanwhile, DMF, which acts as a nonsolvent for PLA at room temperature, remains trapped within the polymer jet. The concentration gradient and the incompatibility between PLA and DMF induce phase separation, resulting in the formation of polymer-rich and solvent-rich regions. After complete evaporation of DMF, the solvent-rich regions transform into pores, resulting in a porous internal structure. This phenomenon has also been reported during electrospinning processes, where the presence of DMF promotes phase separation due to its role as a nonsolvent for PLA at room temperature.^25–28^ The present study demonstrated that the same nonsolvent-induced phase separation occurs during centrifugal spinning, which allows for faster fiber production compared to electrospinning. In this process, the PLA polymer jet similarly separates into polymer-rich and solvent-rich regions, with the polymer-rich regions solidifying into the fiber structure and the solvent-rich regions eventually forming pores. A schematic illustration of the pore formation mechanism in PLA fibers is shown in Fig. 3.

Nitrogen adsorption/desorption measurements were conducted to quantitatively evaluate the specific surface area of the fabricated PLA fibrous membranes. As shown in Table 1, the specific surface area of the obtained PLA fibrous membranes increased with the increase in the DMF ratio in the spinning solution. Generally, the specific surface area of fibrous membranes tends to increase as the fiber diameter decreases. However, in this study, the specific surface area increased with increasing fiber diameter. This result suggests that the NIPS process promotes the formation of porous structures in the PLA fibers. In addition, surface analysis by SEM images revealed that the PLA_0 fibers exhibit numerous elliptical porous structures with a major axis of approximately 312 nm and a minor axis of approximately 67 nm. However, these structures did not contribute to the specific surface area, as they are shallow pores formed by the breath figure phenomenon. The surface morphology of PLA_5 fibers show smooth, with very few shallow pores distributed sparsely, having an average size of approximately 265 nm. Consequently, the specific surface area of PLA_5 fibers was comparable to that of PLA_0 fibers. On the other hand, PLA_10, PLA_15, and PLA_20 fibers exhibit well-developed porous structures with depth, with average pore sizes of approximately 201 nm (minor axis: 67 nm), 430 nm (minor axis: 134 nm), and 630 nm (minor axis: 160 nm), respectively. The results of each pore size are summarized in Table 1. The result indicates that the structures contributing to the specific surface area are porous structures composed of deep pores extending throughout the interior of the fibers, while shallow pores do not contribute significantly. However, the true specific surface area of the fabrous membranes formed from these porous fibers cannot be fully evaluated by gas adsorption analysis alone. Therefore, further evaluation using mercury intrusion porosimetry or gas permeation methods will be required.

**Table 1:** Specific surface area and pore size of the PLA fibers fabricated using solvents with different CHCl_3_/DMF ratios

Sample	PLA_0	PLA_5	PLA_10	PLA_15	PLA_20
Surface area (m^2^ g^−1^)	2.3	2.3	4.9	6.6	6.7
Shallow pore^a^ (nm)	312/67	265/265	—	—	—
Deep pore^a^ (nm)	—	—	201/67	430/134	630/160

a pore size: major axis/minor axis.

### 3.2 Crystallinity and thermal properties of PLA fibers

**Fig. 4:**
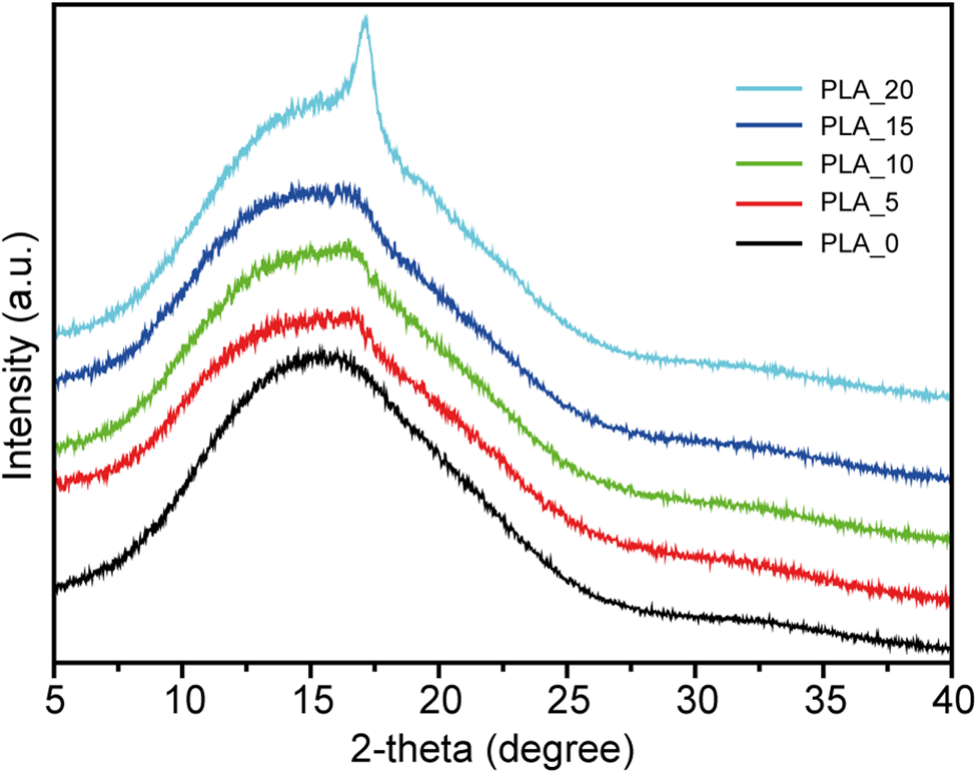
XRD patterns of the PLA fibers obtained from various CHCl_3_/DMF mixture solutions.

**Fig. 5:**
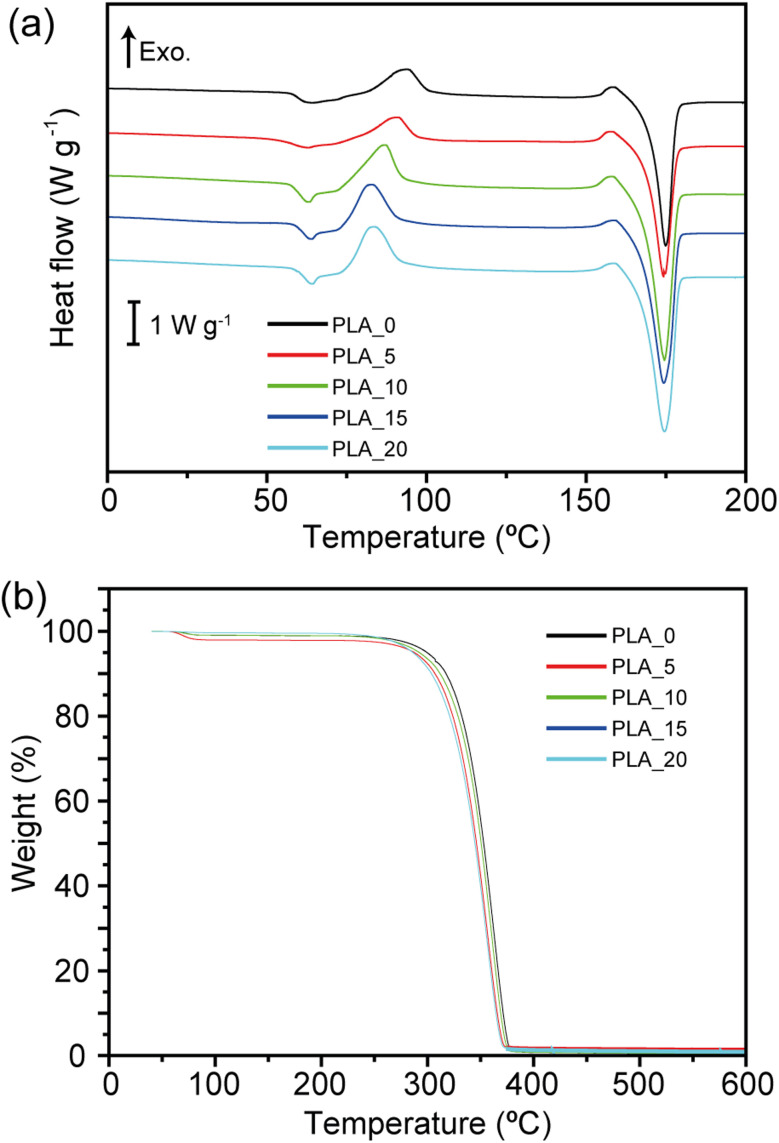
(a) DSC and (b) TGA curves of the PLA fibers prepared from 10 wt% PLA solutions with different weight ratios of CHCl_3_/DMF.

The crystallinity of PLA fibers prepared from different spinning solutions was characterized using XRD. Fig. 4 presents the XRD patterns of PLA fibers spun from PLA solutions with various CHCl_3_/DMF weight ratios. PLA fibrous membranes prepared from PLA solutions with a weight ratio of 100/0 exhibited only a broad peak corresponding to an amorphous region in the XRD spectrum. In contrast, sharp peaks indicative of crystalline regions were absent, suggesting minimal crystalline domain formation. The amorphous nature of these PLA fibers can be attributed to the rapid evaporation rate of the spinning solvent, which limits the time available for crystallization. This fast solvent removal prevents polymer chains from arranging into an ordered crystalline structure. Similarly, PLA fibrous membranes prepared from PLA solutions with CHCl_3_/DMF weight ratios of 95/5, 90/10, and 85/15 also exhibited broad peaks associated with amorphous regions, indicating that minor variations in DMF content do not significantly affect crystallinity. However, when the DMF content reached 20%, a distinct transformation was observed. A sharp peak emerged in the XRD patterns of PLA_20 samples at 17.1°, corresponding to the crystalline (110) and (200) planes of the α-form.^20^ The enhanced crystalline structure could be attributed to phase separation induced by NIPS, which promotes crystallization.^29^ During the spinning process, CHCl3 evaporates faster than DMF due to its lower boiling point. The increased DMF ratio in the spinning solution facilitated phase separation, leading to greater crystallinity. This suggests that adjusting the solvent ratio is crucial for tuning the crystallinity of PLA fibers, which may significantly influence their mechanical strength, thermal properties, and oil adsorption performance. The thermal behavior of PLA fibers was characterized using DSC and TGA, as shown in Fig. 5. The obtained thermal properties, including glass transition temperature (*T*_g_), cold crystallization temperature (*T*_cc_), cold crystallization enthalpy (Δ*H*_cc_), melting temperature (*T*_m_), thermal decomposition temperature (*T*_d_), melting enthalpy (Δ*H*_m_), and crystallinity (*X*), are summarized in Table 1. The *T*_g_ and *T*_m_ values of PLA fibers remained unchanged across different CHCl_3_/DMF weight ratios, indicating that the fundamental polymer structure was not significantly affected by the solvent composition. However, an increase in the DMF ratio led to a decrease in cold crystallization temperature. Additionally, the cold crystallization peaks shifted to lower temperatures and became narrower as the DMF ratio increased. This implies that the presence of DMF in the spinning solution facilitates crystallization at lower temperatures, likely due to enhanced molecular mobility during thermal treatment. The shift in the cold crystallization peak might be attributed to the presence of a semi-ordered amorphous phase. As previously reported, a high evaporation rate of CHCl_3_ impedes complete crystallization, forming a semi-ordered amorphous phase that can be easily transformed into a crystalline phase through thermal aging.^30^ Although XRD patterns did not explicitly confirm changes in crystallinity, DSC results indicated an increase in crystallinity with a higher DMF ratio in the spinning solution, as shown in Table 2. PLA fibers prepared using only CHCl_3_ had a *X* of 15.81 ± 3.47%, whereas an increased DMF content led to crystallinity levels of approximately 24–26% in PLA/CHCl_3_/DMF systems. These findings demonstrate that the crystallinity of centrifugally spun PLA fibers can be effectively enhanced by modifying the solvent composition. In contrast, *T*_d_ values of all samples showed no significant differences, as the chemical composition remained unchanged. This suggests that the thermal stability of PLA fibers is primarily governed by the intrinsic properties of the polymer rather than the solvent ratio. However, the increased crystallinity observed with higher DMF content may contribute to improved mechanical stability, which is advantageous for various applications. These findings highlight the critical role of solvent selection in tailoring the structural and thermal properties of PLA fibers. Future research should further investigate the mechanical properties of these fibers concerning their crystallinity and explore their potential applications in oil adsorption and biodegradable material development. Additionally, further studies should analyze the long-term stability and recyclability of PLA fibrous membranes to assess their suitability for sustainable applications. Moreover, investigating the impact of various solvent compositions on the mechanical strength and flexibility of PLA fibers will provide deeper insights into their practical performance. Evaluating the effect of prolonged exposure to different environmental conditions, including humidity and temperature fluctuations, will further support the development of optimized PLA-based materials for industrial and environmental applications.

**Table 2:** Thermal properties of the PLA fibers obtained from DSC and TGA characterization

Sample	*T*_g_ (°C)	*T*_cc_ (°C)	*T*_m_ (°C)	*T*_d_ (°C)	*X* (°C)
PLA_0	59.4 ± 0.7	93.9 ± 0.3	174.8 ± 0.1	363.6 ± 0.5	15.8 ± 3.5
PLA_5	57.2 ± 2.0	90.4 ± 0.9	174.6 ± 0.3	359.7 ± 2.4	24.6 ± 2.2
PLA_10	60.3 ± 1.4	86.8 ± 0.5	174.5 ± 0.0	360.5 ± 4.1	22.7 ± 2.1
PLA_15	61.4 ± 0.4	82.4 ± 0.4	174.5 ± 0.2	359.6 ± 4.5	26.2 ± 3.2
PLA_20	62.1 ± 0.3	83.0 ± 0.4	174.7 ± 0.4	359.4 ± 6.3	25.3 ± 3.1

### 3.3 Wettability of PLA fibers

**Fig. 6:**
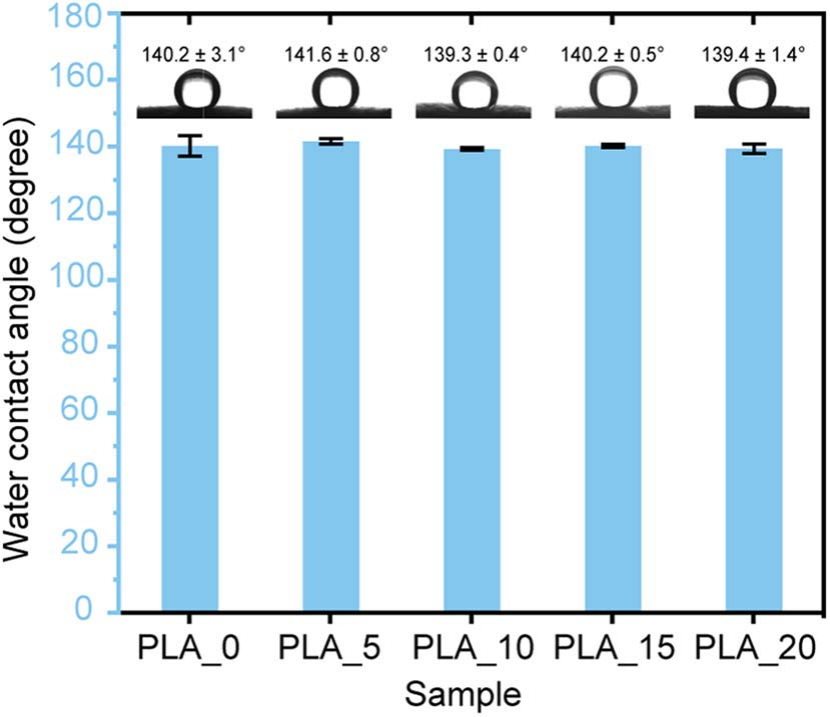
Water contact angle of the prepared PLA fibrous membranes.

Fig. 6 shows the water contact angle values and the photographs of the water droplet on the obtained PLA fibrous membrane. It was found that a significant difference was not observed in the water contact angle between the prepared samples. The obtained PLA fibrous membranes showed high water contact angles from 133.9° to 141.6°. The high hydrophobicity of the PLA fibrous membranes is mainly attributed to the increase in surface roughness. The high surface roughness of the fibrous mats caused the wetting transition from the Young state to the Wenzel state, resulting in a high water contact angle.^31^ On the other hand, when an oil droplet contacted the PLA mats, the oil droplet was rapidly absorbed, and the oil contact angle was nearly 0°, indicating that the prepared PLA fibrous mats are superoleophilic. The low water absorption and high oil sorption rate indicate that they are suitable candidates for oil removal applications.

**Fig. 7:**
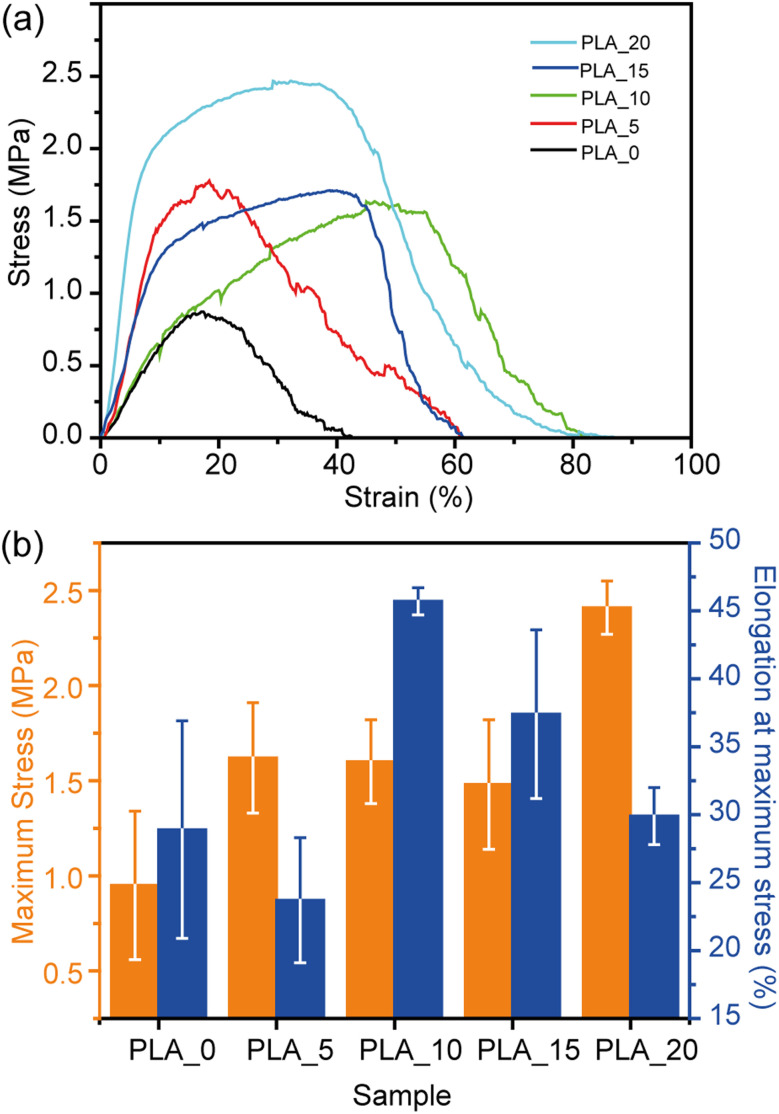
(a) Stress–strain curves and (b) mechanical properties of PLA webs prepared from different PLA solutions.

The mechanical properties of PLA fibrous membranes were evaluated by tensile test, as shown in Fig. 7. Overall, the mechanical strength of the PLA membrane increased with the DMF ratio in the spinning solutions. The increase in fiber diameter or crystallinity might improve the stability of the fibrous membrane. The tensile strength of the PLA fibrous membranes prepared in this work is comparable to PLA fibers fabricated using the electrospinning technique.^32^ The PLA_20 showed the highest mechanical strength with maximum stress and elongation at a maximum stress of 2.41 ± 0.14 MPa and 29.9 ± 2.1%, respectively. The improved mechanical strength of PLA_20 could be suitable for oil absorption applications.^33,34^

### 3.4 Oil absorption

**Fig. 8:**
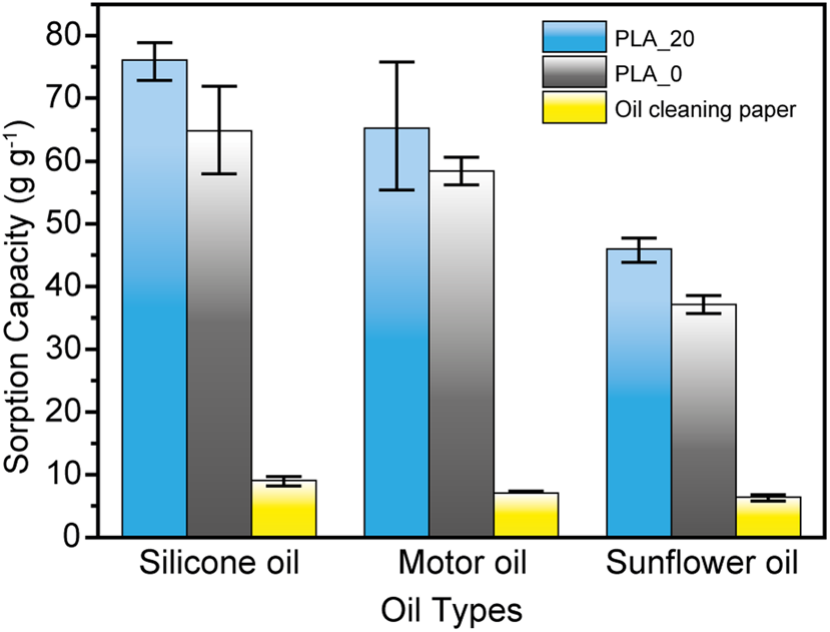
Maximum sorption capacities of the PLA_0, PLA_20 fibrous membranes and an oil cleaning paper for different oils.

The oil sorption capacity of PLA_0, PLA_20, and commercial oil cleaning paper is shown in Fig. 8. The sorption capacities of the PLA_0 for silicone oil, motor oil, and sunflower seed oil were 64.4 ± 7.0, 58.4 ± 2.2, and 37.1 ± 1.4 g g^−1^, respectively. On the other hand, the PLA_20 fibrous membrane showed higher sorption capacities for silicone oil, motor oil, and sunflower seed oil, which were 76.1 ± 2.8, 65.3 ± 10.5, and 46.0 ± 1.8 g g^−1^, respectively. This result might be considered as a higher surface area of the PLA fibrous membrane improved the sorption capacity. A higher surface roughness of PLA_20 fibers could lead to more remaining oil. The PLA fibrous membranes exhibited higher oil sorption capacities than the commercial oil cleaning paper, which are 9.1 ± 0.6, 6.8 ± 0.3, and 6.4 ± 0.4 g g^−1^, respectively. The result indicates that the PLA_20 possesses approximately 7–9 times higher oil adsorption capacity than commercial oil cleaning paper for silicone oil, motor oil, and sunflower oil. Although the data are not shown here, it was also confirmed that PLA fibrous membranes exhibit minimal reduction in adsorption capacity even after repeated use, demonstrating excellent recyclability. These findings suggest that PLA fibrous membranes not only have great potential as highly efficient oil-absorbing materials but also contribute to reducing environmental impact. This superior performance of PLA fibrous membranes is likely due to their deep pore (void) volume and interconnected porous structure, which can accommodate larger amounts of oil compared to the relatively compact structure of commercial oil-cleaning paper. At the CHCl_3_/DMF ratio of 80/20, PLA_20, the PLA fibrous membrane exhibits uniform pore size distribution, enhanced hydrophobicity, increased surface area, and excellent mechanical stability, leading to the highest oil adsorption efficiency within this system. The increase in DMF content contributed to a higher surface area and modifications in pore morphology, which enhanced capillary effects and subsequently improved oil sorption performance. However, the relationship between surface area or pore depth and oil adsorption capacity was not strictly proportional. Instead, the dominant factor appears to be the effective surface area at the micron scale, formed by fiber entanglement throughout the fibrous membrane, which plays a crucial role in oil adsorption.

In addition, the sorption capacities were also related to the viscosity of the three kinds of oils, as shown in Table S1.† The high viscosity can improve the sorption capacity because oil can adhere strongly to the sorbent surface, increasing the oil absorption performance.

Table 3 shows a comparison of the oil absorption capacity of porous PLA fibers prepared in this study compared to PLA prepared by different spinning techniques in previous reports. It can be seen that the PLA fibrous membrane prepared in this work showed higher oil sorption performance compared to that of other centrifugally spun PLA fibers. In contrast to electrospun PLA fibers, PLA_20 exhibits lower oil sorption performance due to the bigger fiber diameter, resulting in lower surface area. When evaluating the production efficiency of fiber and oil adsorption capacity of the PLA fibrous membrane using the FOM, it can be confirmed that the PLA fibrous membrane performs significantly better, showing approximately 40–130 times greater efficiency compared to conventional electrospinning (ES) methods (Table 3). This result is attributed to the production efficiency of centrifugal spinning (CS), which is 13.3 g min^−1^, far exceeding that of ES (approximately 0.1–0.3 g min^−1^),^35–38^ thus leading to a higher FoM evaluation. Therefore, it can be concluded that centrifugal spinning is a feasible approach for preparing PLA fibers for oil removal applications.

**Table 3:** Comparison of oil sorption performance of PLA fibers prepared in this work and published studies

Spinning technique	Oil type	Oil sorption capacity (g g^−1^)	FoM	Reference
CS	Silicone oil	76.1	13.3	This work
CS	Silicone oil	49.8	n.a.	34
ES	Silicone oil	*ca*. 270	0.2	35
ES	Mineral oil	104 ± 5.4	0.2	36
ES	Vacuum pump oil	42.38	0.1	37
ES	Soybean oil	26.8 ± 0.1	0.3	38

## 4. Conclusion

In conclusion, porous, highly hydrophobic, and superoleophilic PLA fibers were successfully fabricated using a high-productivity centrifugal spinning technique. The porous structure of the PLA fibers was controlled by adjusting the ratio of DMF and CHCl_3_ in the spinning solution. PLA fibers produced from a solution with a CHCl_3_/DMF ratio of 80/20 exhibited a fiber diameter of 3.50 ± 1.60 μm and a specific surface area of 6.7 m^2^ g^−1^. The oil sorption capacities for silicone oil, motor oil, and sunflower seed oil reached 76.1 ± 2.8, 65.3 ± 10.5, and 46.0 ± 1.8 g g^−1^, respectively, demonstrating that the fabricated porous PLA fibers have great potential as environmentally friendly oil absorbents. However, several challenges remain. Achieving uniform fiber diameters and precise control is difficult, as increasing the DMF ratio leads to larger fibers, which may reduce specific surface area and oil absorption performance. Additionally, the nitrogen adsorption method may not fully reflect the impact of deep pores, requiring further evaluation using techniques like mercury intrusion porosimetry. Improving mechanical strength and durability, optimizing crystallinity and molecular orientation, and combining PLA with other biodegradable materials may enhance the fibers. Further evaluation of PLA's degradation rate and environmental impact, particularly in marine environments, is necessary. Moreover, large-scale industrial applications of centrifugal spinning will require further optimization to improve production efficiency. The fabrication process developed in this study can be applied to other polymers, and the development of porous fibers based on different systems is anticipated.

## Data availability

The data supporting this article have been included as part of the ESI† and in the main manuscript.

## Conflicts of interest

The authors declare no conflict of interest.

## Supplementary Material

RA-015-D4RA08727E-s001
