# Supplementary material for: Porous polylactic acid fibers synthesized by centrifugal spinning with phase separation for oil removal application
Source: RSC Adv. 2025 Apr 15;15(15):11749–58. doi: 10.1039/d4ra08727e (PMC11997648; doi:10.1039/d4ra08727e)
Supplement: RA-015-D4RA08727E-s001 [file RA-015-D4RA08727E-s001.pdf]

Supplementary Information (SI) for RSC Advances.
This journal is © The Royal Society of Chemistry 2025

Supporting Information

Porous polylactic acid fibers by centrifugal spinning with phase separation for oil removal application

Kenji Kinashi[1]*, Masaki Negoro[2], Hoan Ngoc Doan[3,4]*, Phu Phong Vo[4,5], Khanh Van Thi Khuat[6], Wataru Sakai[1], Naoto Tsutsumi[1]

[1] Faculty of Materials Science and Engineering, Kyoto Institute of Technology, Matsugasaki, Sakyo, Kyoto 606-8585, Japan.
[2] Master's Program of Innovative Materials, Kyoto Institute of Technology, Matsugasaki, Sakyo, 606-8585 Kyoto, Japan
[3] Tissue Engineering and Regenerative Medicine Laboratory, School of Biomedical Engineering, International University.
[4] Vietnam National University Ho Chi Minh City, Vietnam.
[5] Faculty of Chemistry, University of Science, Ho Chi Minh City, Vietnam.
[6] Doctor's Program of Materials Chemistry, Graduate School of Science and Technology, Kyoto Institute of Technology, Matsugasaki, Sakyo, Kyoto 606-8585, Japan.

Corresponding author emails: kinashi@kit.ac.jp; dnhoan@hcmiu.edu.vn

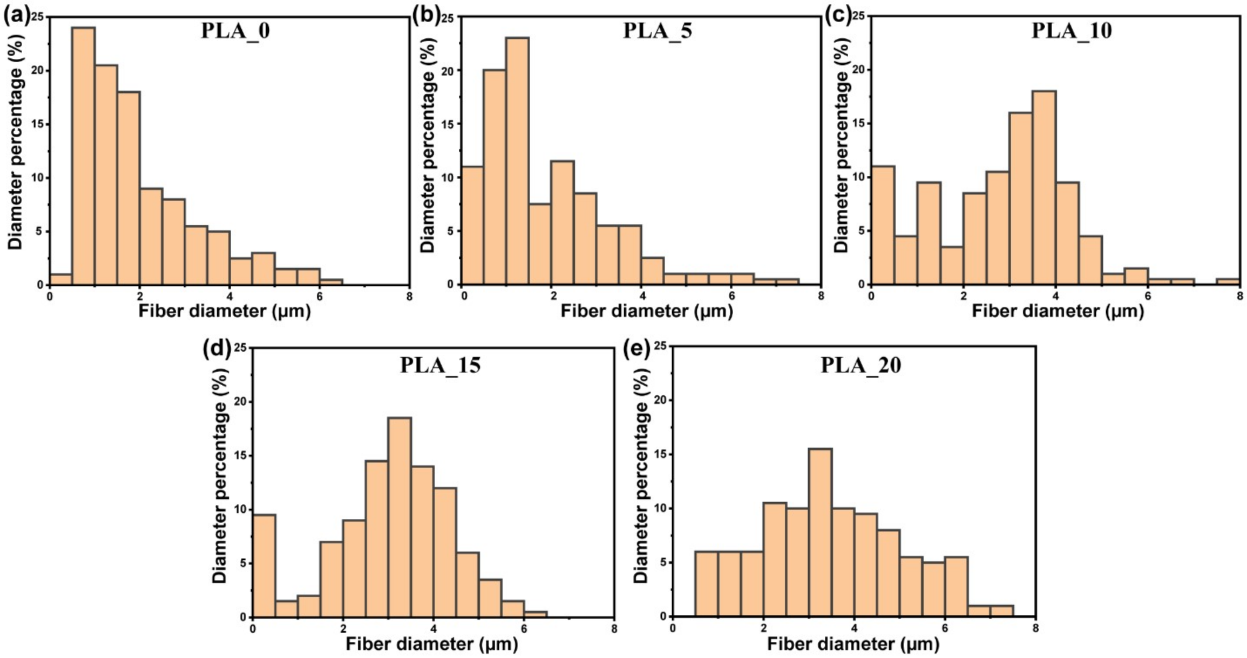

Figure S1. Fiber diameter distributions of the PLA fibers prepared from 10 wt% PLA solutions with varying DMF ratios.

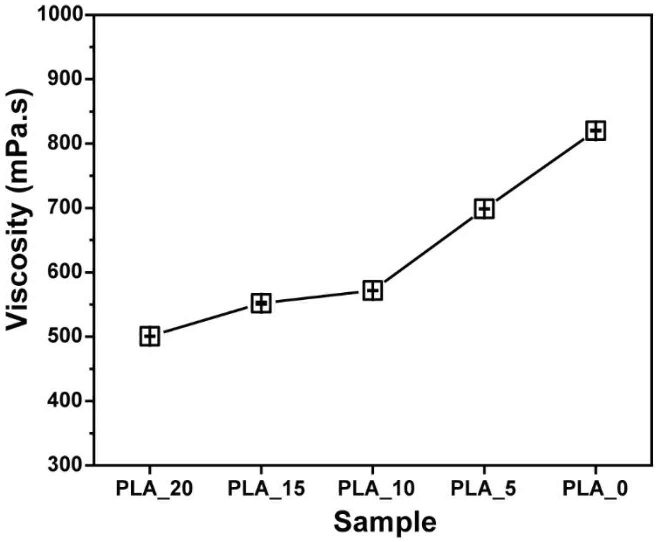

Figure S2. Viscosity of PLA solutions with varying $CHCl_3$/DMF ratios.

Table S1. Viscosity of the oils used for the oil absorption test.

Oil type	Viscosity (mPa s)
Silicone oil	421
Motor oil	207
Sunflower oil	70
